# Body mass index in early adulthood and colorectal cancer risk for carriers and non-carriers of germline mutations in DNA mismatch repair genes

**DOI:** 10.1038/bjc.2011.172

**Published:** 2011-05-10

**Authors:** A K Win, J G Dowty, D R English, P T Campbell, J P Young, I Winship, F A Macrae, L Lipton, S Parry, G P Young, D D Buchanan, M E Martínez, E T Jacobs, D J Ahnen, R W Haile, G Casey, J A Baron, N M Lindor, S N Thibodeau, P A Newcomb, J D Potter, L Le Marchand, S Gallinger, J L Hopper, M A Jenkins

**Affiliations:** 1 Centre for Molecular, Environmental, Genetic and Analytic Epidemiology, The University of Melbourne, Melbourne School of Population Health, Level 3, 207 Bouverie Street, Parkville, Victoria 3010, Australia; 2 Cancer Epidemiology Centre, Cancer Council Victoria, Carlton South, Victoria, Australia; 3 Epidemiology Research Program, American Cancer Society, Atlanta, GA, USA; 4 Cancer Prevention Program, Fred Hutchinson Cancer Research Centre, Seattle, WA, USA; 5 Familial Cancer Laboratory, Queensland Institute of Medical Research, Herston, Queensland, Australia; 6 Adult Clinical Genetics, The University of Melbourne, Parkville, Victoria, Australia; 7 Department of Colorectal Medicine and Genetics, The Royal Melbourne Hospital, Parkville, Victoria, Australia; 8 Ludwig Institute for Cancer Research, The Royal Melbourne Hospital, Parkville, Victoria, Australia; 9 New Zealand Familial Gastrointestinal Cancer Registry, Auckland City Hospital, Auckland, New Zealand; 10 Department of Gastroenterology, Middlemore Hospital, Auckland, New Zealand; 11 Flinders Centre for Cancer Prevention and Control, Flinders University, Adelaide, South Australia, Australia; 12 Arizona Cancer Centre, University of Arizona, Tucson, AZ, USA; 13 Mel and Enid Zuckerman College of Public Health, University of Arizona, Tucson, AZ, USA; 14 Denver VA Medical Center and University of Colorado Denver School of Medicine, Denver, CO, USA; 15 Department of Preventive Medicine, University of Southern California, Los Angeles, CA, USA; 16 Department of Medicine and Department of Community and Family Medicine, Dartmouth Medical School, Lebanon, NH, USA; 17 Department of Medical Genetics, Mayo Clinic, Rochester, MN, USA; 18 Cancer Research Center of Hawaii, University of Hawaii, Honolulu, HI, USA; 19 Cancer Care Ontario, Toronto, Ontario, Canada

**Keywords:** body mass index, colorectal cancer, mismatch repair gene

## Abstract

**Background:**

Carriers of germline mutations in DNA mismatch repair (MMR) genes have a high risk of colorectal cancer (CRC), but the modifiers of this risk are not well established. We estimated an association between body mass index (BMI) in early adulthood and subsequent risk of CRC for carriers and, as a comparison, estimated the association for non-carriers.

**Methods:**

A weighted Cox regression was used to analyse height and weight at 20 years reported by 1324 carriers of MMR gene mutations (500 *MLH1*, 648 *MSH2*, 117 *MSH6* and 59 *PMS2*) and 1219 non-carriers from the Colon Cancer Family Registry.

**Results:**

During 122 304 person-years of observation, we observed diagnoses of CRC for 659 carriers (50%) and 36 non-carriers (3%). For carriers, the risk of CRC increased by 30% for each 5 kg m^–2^ increment in BMI in early adulthood (hazard ratio, HR: 1.30; 95% confidence interval, CI: 1.08–1.58; *P*=0.01), and increased by 64% for non-carriers (HR: 1.64; 95% CI: 1.02–2.64; *P*=0.04) after adjusting for sex, country, cigarette smoking and alcohol drinking (and the MMR gene that was mutated in carriers). The difference in HRs for carriers and non-carriers was not statistically significant (*P*=0.50). For *MLH1* and *PMS2* (MutL*α* heterodimer) mutation carriers combined, the corresponding increase was 36% (HR: 1.36; 95% CI: 1.05–1.76; *P*=0.02). For *MSH2* and *MSH6* (MutS*α* heterodimer) mutation carriers combined, the HR was 1.26 (95% CI: 0.96–1.65; *P*=0.09). There was no significant difference between the HRs for MutL*α* and MutS*α* heterodimer carriers (*P*=0.56).

**Conclusion:**

Body mass index in early adulthood is positively associated with risk of CRC for MMR gene mutation carriers and non-carriers.

Lynch syndrome, historically known as hereditary non-polyposis colorectal cancer (Jass, 2006), refers to colorectal and other cancers caused by germline mutations in DNA mismatch repair (MMR) genes *MLH1*, *MSH2*, *MSH6* and *PMS2* (Vasen *et al*, 1999). Approximately 1 in 3000 people in the general population carry a mutation in an MMR gene (Dunlop *et al*, 2000). These MMR gene mutation carriers are at substantially increased risk of colorectal cancer (CRC) with an estimated cumulative risk to age 70 years between 40% and 70% depending on the carrier's sex and the MMR gene that is mutated (Chen *et al*, 2006; Jenkins *et al*, 2006; Senter *et al*, 2008; Baglietto *et al*, 2010). Physical characteristics or environmental exposures of the mutation carriers could also modify their risks of developing CRC (Jenkins *et al*, 2007). Identifying modifiers of CRC risk for carriers of MMR gene mutations is important for understanding carcinogenesis. Identifying potentially protective or harmful and avoidable risk factors also creates opportunities for mutation carriers to reduce their risks of life-threatening diseases.

Previous studies have provided evidence for the existence of modifiers of CRC risk for MMR gene mutation carriers. For example, CRC risk for carriers is positively associated with alcohol consumption (Watson *et al*, 2004) and negatively associated with fruit consumption and dietary fibre intake (Diergaarde *et al*, 2007). For smoking, CRC risk for carriers is positively associated with current smoking (Diergaarde *et al*, 2007; Pande *et al*, 2010), but negatively or not associated with former smoking (Diergaarde *et al*, 2007; Pande *et al*, 2010). However, the association between body size and CRC risk for MMR gene mutation carriers is yet to be established.

One CRC risk factor for people in the general population is body size, a collective term for body mass index (BMI), waist circumference and height (Dai *et al*, 2007; Larsson and Wolk, 2007; Moghaddam *et al*, 2007; Renehan *et al*, 2008). Previous case–control studies of CRC cases with a family history (therefore, likely to be enriched for MMR gene mutation carriers) compared with population-based controls who were unselected for family history, have shown that current BMI is positively associated with CRC risk (Slattery *et al*, 2003; Campbell *et al*, 2007). However, studies that have examined the association between current BMI and the occurrence of tumours with microsatellite instability (MSI), a common characteristic of CRCs caused by MMR defects, have reported no statistically significant associations with this form of disease (Slattery *et al*, 2000; Campbell *et al*, 2010). One study that examined the association between BMI and colorectal adenoma risk for MMR gene mutation carriers observed a positive association for males but not females (Botma *et al*, 2010).

Identifying an association of BMI in early adulthood on subsequent cancer risk would be important for MMR gene mutation carriers who learn their mutation status as adults in their 20s, an increasingly common occurrence because of increased systematic testing in the population, with the consequent opportunity to reduce their risk of disease. In this study, we estimated an association between BMI at age 20 years and CRC risk for MMR gene mutation carriers. As a comparison, we also estimated the association for non-carriers to investigate whether BMI has a differential effect on risk for CRC by mutation status.

## Materials and methods

### Recruitment

Subjects comprised carriers and non-carriers of pathogenic mutations (see below) in the MMR genes *MLH1*, *MSH2*, *MSH6* and *PMS2*, recruited and genetically characterised by the Colon Cancer Family Registry (Colon CFR). Study designs and recruitments for the Colon CFR can be found at http://epi.grants.cancer.gov/CFR/ and have been published in detail (Newcomb *et al*, 2007). Probands were either a recently diagnosed CRC case reported to a population-complete cancer registry or an attendee at a family cancer clinic. They were recruited between 1997 and 2007 and ascertained from family cancer clinics from Australia (Melbourne, Adelaide, Perth, Brisbane and Sydney), New Zealand (Auckland) and the United States (Mayo Clinic, Rochester, Minnesota and Cleveland) or from population-complete cancer registries in the United States (Puget Sound, Washington State; the State of Minnesota; Los Angeles, California; Arizona; Colorado; New Hampshire; North Carolina; and Hawaii), Australia (Victoria) and Canada (Ontario).

Probands were asked for permission to contact their relatives to seek their enrolment in the Colon CFR. For probands ascertained from family cancer clinics, there were pre-specified rules consistent across recruiting centres governing which relatives were to be approached for recruitment (Newcomb *et al*, 2007). For probands ascertained from population-complete cancer registries, first-degree relatives were recruited at all centres and in some centres, recruitment extended to more distant relatives. Written informed consent was obtained from all study participants, and the study protocol was approved at each Colon CFR centre.

### Data collection

At recruitment, baseline information on demographics, personal characteristics, personal and family history of cancer, cancer screening history, history of polyps, polypectomy and other surgery were obtained from all participants. Participants were followed-up approximately 5 years after baseline to update all this information. The questionnaires are available from http://cfrisc.georgetown.edu/isc/ dd.questionnaires.do. Reported cancer diagnoses and age at diagnoses were confirmed, where possible, using pathology reports, medical records, cancer registry reports and/or death certificates. Blood samples and tumour tissues were collected for genetic testing. The present study was based on all available baseline and follow-up data.

Self-reported height and weight at approximately age 20 years were collected using standardised personal interviews (University of Southern California Consortium), telephone interviews (University of Southern California Consortium, Fred Hutchinson Cancer Research Centre and University of Melbourne) or mailed questionnaires (University of Hawaii, Cancer Care Ontario and Mayo Clinic).

### Mutation screening and testing

Testing for *MLH1*, *MSH2*, *MSH6* and *PMS2* mutations was performed for all case probands ascertained from family cancer clinics and for all probands from population-based ascertainment who had a colorectal tumour displaying evidence of impaired MMR function as evidenced by either MSI, or by lack of MMR protein expression by immunohistochemistry. Mutation testing was performed by Sanger sequencing or denaturing high-pressure liquid chromatography, followed by confirmatory DNA sequencing. Large insertion and deletion mutations were detected by multiplex ligation-dependent probe amplification according to the manufacturer's instructions (MRC Holland, Amsterdam, The Netherlands) (Southey *et al*, 2005; Newcomb *et al*, 2007; Senter *et al*, 2008). All participants who donated a blood sample, and who were relatives of probands with a pathogenic mutation, underwent testing for the same mutation identified in the proband.

### Study sample

For this study, we included male and female probands and their participating relatives who were confirmed carriers and confirmed non-carriers of an MMR gene mutation. In total, 1468 carriers and 1365 non-carriers were identified. Of these, 144 (10%) carriers and 146 (11%) non-carriers were excluded due to missing data on height or weight at age 20 years, leaving a total of 1324 carriers (252 from population-based sources) and 1219 non-carriers (109 from population-based sources) for the analysis. Excluded subjects did not differ in baseline characteristics from subjects who entered into the analyses (age, sex, country and gene mutated) (data not shown).

We were unable to analyse current BMI in this study, as it was not available 1–2 years prior to diagnosis or censored age for 833 subjects (636 carriers and 197 non-carriers), which is 33% of the total sample size. This was composed of 450 CRC cases (425 carriers and 25 non-carriers), 215 subjects (140 carriers and 75 non-carriers) with another cancer and 164 subjects with a prior polypectomy (70 carriers and 94 non-carriers) and 4 subjects (1 carrier and 3 non-carriers) who did not report current weight at last contact.

### Definitions

A pathogenic mutation was defined to be a variant that was predicted to result in a stop codon, a frameshift mutation, a large insertion or deletion, or a missense mutation previously reported in the scientific literature to be pathogenic. Age at CRC was the age at first diagnosis of CRC. Height and weight were defined as self-reported height and weight at age 20 years, respectively. BMI at age 20 years was calculated as weight as reported at age 20 years in kilograms divided by height in metres squared (kg m^–2^). Body mass index was categorised as ⩽18.49 kg m^–2^ (underweight), 18.50–24.99 kg m^–2^ (normal weight), 25.00–29.99 kg m^–2^ (overweight) or ⩾30.00 kg m^–2^ (obese) according to World Health Organization criteria (World Health Organization, 1998). T and N staging of CRC was categorised according to the American Joint Committee on Cancer staging (Edge *et al*, 2009).

### Statistical analysis

Mismatch repair gene mutation carriers and non-carriers were treated as a cohort from birth. A proportional Cox regression model with age as the time scale was used to estimate hazard ratios (HR) relating BMI to CRC risk for carriers and non-carriers. Time at risk started at birth and ended at age of diagnosis of cancer of any site, polypectomy, death or last contact, whichever occurred first. The rationale for censoring at diagnosis of any cancer was that the resultant treatment and surveillance might alter risk of subsequent cancers, including CRC and this would introduce bias if the first cancer was associated with body size. Similarly, as polypectomy may reduce CRC risk, censoring at time of polypectomy was also required to avoid potential bias. Of the total 1324 mutation carriers, observation time ended at age of diagnosis for 659 CRC cases, and was censored at age of polypectomy for 173 subjects, at age of diagnosis of another cancer for 144 subjects, at age of death for 4 subjects and at age of last contact for 344 subjects. Of the total 1219 non-carriers, time of observation ended at the age of diagnosis for 36 CRC cases, at age of polypectomy for 176 subjects, at age of diagnosis of another cancer for 101 subjects, at age of death for 14 subjects and at age of last contact for 892 subjects.

Since some subjects were ascertained because they were from multiple-case CRC families, and CRC cases were preferentially tested for MMR gene mutations, the selection of subjects was not random with respect to disease status. To adjust for this non-random ascertainment, we used the weighted cohort approach described by Antoniou *et al* (2005), which has been used for modifier studies of cancer risk for carriers of rare genetic mutations (e.g., Andrieu *et al*, 2006; Antoniou *et al*, 2008; Win *et al*, 2011). A simulation study of this approach (Antoniou *et al*, 2005) showed that allowing for non-random sampling of subjects by using rates from an external referent population removed bias when the external rates were correctly specified, and reduced bias if the external rates were not completely accurate.

Age-specific incidence rates of CRC for MMR gene mutation carriers were previously estimated as described in detail by Pande *et al* (2010). These age-specific incidence rates were used to calculate sampling fractions to weight the proportion of affected and unaffected carriers in each age stratum, so that the proportion of affected carriers in each age group equalled the population proportions (Supplementary Table S1).

Due to the rarity of mutations in the population, age-specific incidence rates for non-carriers were assumed to equal those for the general population. Data on age-specific (5-year intervals) population cancer incidence rates were obtained from the American Cancer Society Cancer Facts and Figures 2008 (American Cancer Society, 2008). Using these age-specific incidence rates, we calculated sampling fractions to weight the proportion of affected and unaffected non-carriers in each age stratum.

Body mass index was fitted as a continuous variable and as a categorical variable based on WHO BMI classification (World Health Organization, 1998). In order to determine whether a non-linear association with a continuous variable was a superior fit compared with a linear model, we fitted and compared fractional polynomial models. Models were compared using Wald's tests. The proportional hazards assumption was tested by examining the relationship between the scaled Schoenfeld residuals and survival time.

To control for potential confounders present at the age at which BMI was reported (i.e., 20 years old), we adjusted for (i) sex, (ii) country of recruitment, (iii) cigarette smoking at age 20 (never, ever) and (iv) alcohol drinking at age 20 (ever, never) in both carriers and non-carriers, and further adjusted for (v) specific MMR gene that was mutated in carriers. Interactions between (i) mutation carrier status and BMI, (ii) sex and BMI and (iii) the specific MMR gene mutated and BMI were tested by adding an interaction term in a multivariable model. We did not adjust for current BMI because of the well-known bias introduced by adjusting for variables that are in the causal pathway or partly caused by the exposure of interest. The associations between BMI and CRC risk were estimated separately for carriers of mutation in each MMR gene, and carriers of mutations in the MutL*α* heterodimer (*MLH1* and *PMS2*) and MutS*α* heterodimer (*MSH2* and *MSH6*) genes.

Two sensitivity analyses were conducted: (i) to determine whether unverified self-reported CRC cases or cases reported by relatives influenced the associations with BMI, we performed analyses after excluding these cases and (ii) to determine whether censoring at polypectomy influenced the associations with BMI, we performed analyses of colorectal neoplasia that is CRC and colorectal polyps combined, as the outcome.

We applied the Huber–White robust variance estimation by clustering on family membership to allow for any correlation of risk between family members (Rogers, 1993; Williams, 2000). All statistical tests were two sided and, following convention, statistical significance for testing a predetermined null hypothesis was set at *P*<0.05. All statistical analyses were performed using Stata 10.0 (StataCorp, 2007).

## Results

The study comprised 1324 carriers (737 females) of MMR gene mutations (500 in *MLH1*, 648 in *MSH2*, 117 in *MSH6* and 59 in *PMS2*) from 498 families contributing a total of 58 868 person-years, of which 659 (50%) were diagnosed with CRC, and 1219 non-carriers (712 females) from 287 families contributing 63 436 person-years, of which 36 (3%) were diagnosed with CRC. Of all participants, 839 carriers and 983 non-carriers (72%) were recruited in Australia or New Zealand, 367 carriers and 199 non-carriers (22%) in the United States and 118 carriers and 37 non-carriers (6%) in Canada. Baseline characteristics of the study subjects are summarised in Table 1.

Of the 659 CRC cases in carriers, 595 (90%) were confirmed by pathology report or review, SEER or other cancer registries, or hospital or clinical records and 64 (10%) were unconfirmed reports by self or relatives (Table 2). The mean age at CRC diagnosis was 44 (s.d.: 11) years for carriers and 57 (s.d.: 15) years for non-carriers. The mean reported BMI at age 20 years was 22.7 (s.d.: 3.9) kg m^–2^ for carriers and 22.4 (s.d.: 3.7) kg m^–2^ for non-carriers. There were no significant differences in mean BMI or the distributions of mutated MMR genes by country (data not shown).

Table 3 shows that carriers who were obese (⩾30 kg m^–2^) had an approximately two-fold (HR: 2.35; 95% confidence interval, CI: 1.30–4.23) increased risk of CRC compared with carriers who had normal BMI (18.5–24.99 kg m^–2^). Colorectal cancer risk for carriers was estimated to be 30% higher for each increment of 5 kg m^–2^ in BMI (adjusted HR: 1.30; 95% CI: 1.08–1.58; *P*=0.01). The corresponding HR for female carriers was 1.28 (95% CI: 0.98–1.69; *P*=0.08) and for male carriers was 1.33 (95% CI: 1.01–1.76; *P*=0.04); there was no statistically significant evidence that these HRs were different between men and women (*P*=0.97).

The HR per 5 kg m^–2^ in BMI was 1.20 (95% CI: 0.99–1.44; *P*=0.06) for right-sided colon cancers; 1.03 (95% CI: 0.84–1.27; *P*=0.76) for left-sided colon and rectosigmoid cancers; and 1.31 (95% CI: 0.84–2.05; *P*=0.24) for rectal cancers. When a fractional polynomial model was fitted, there was no evidence that the (log) HR was non-linear (*P*=0.72).

Table 4 presents separate results for carriers of mutations in each MMR gene. There was a positive association between BMI and CRC risk for *MLH1* and *PMS2* (MutL*α* heterodimer) mutation carriers combined (HR: 1.36; 95% CI: 1.05–1.76; *P*=0.02). The HR for *MSH2* and *MSH6* (MutS*α* heterodimer) mutation carriers combined was 1.26 (95% CI: 0.96–1.65; *P*=0.09), and not statistically different from the HR for MutL*α* heterodimer mutation carriers (*P*=0.56).

For non-carriers of MMR gene mutations, CRC risk was estimated to be 64% higher for each increment of 5 kg m^–2^ in BMI (adjusted HR: 1.64; 95% CI: 1.02–2.64; *P*=0.04). The corresponding HR for female carriers was 1.40 (95% CI: 0.67–2.91; *P*=0.37) and for male carriers was 1.82 (95% CI: 0.93–3.53; *P*=0.08); the difference between sexes was not statistically significant (*P*=0.32). When a fractional polynomial model was fitted, there was no evidence that the (log) HR was non-linear (*P*=0.88).

The HRs for carriers and non-carriers were not statistically different (interaction between mutation status and BMI; *P*=0.50).

Sensitivity analyses showed that (i) after excluding unverified self-reported cases or cases reported by relatives, the adjusted HR for each 5 kg m^–2^ increment in BMI was 1.37 (95% CI: 1.13–1.66; *P*=0.002) for carriers and 1.65 (95% CI: 0.97–2.80; *P*=0.06) for non-carriers and (ii) defining the outcome as colorectal neoplasia (either CRC or polyp), the adjusted HR for each 5 kg m^–2^ increment in BMI was 1.20 (95% CI: 1.00–1.44; *P*=0.05) for carriers and 1.27 (95% CI: 0.96–1.66; *P*=0.09) for non-carriers.

## Discussion

We have shown that greater BMI at age 20 years is associated with a higher risk of CRC in later life for both carriers and non-carriers of pathogenic germline mutations in MMR genes. For MMR mutation carriers, we found no statistically significant differences between the strengths of association for men and women, and for carriers of mutations in MutL*α* or MutS*α* heterodimer genes. These results suggest that BMI in early adulthood is a potential modifier of CRC risk in later life for MMR gene mutation carriers as a whole.

Our study was based on 1324 carriers and 1219 non-carriers of a confirmed pathogenic mutation in any of four MMR genes, making it the largest study to date of this issue. To overcome bias when subjects are selected on the basis of phenotype, we used a weighted cohort approach (Antoniou *et al*, 2005), which has been successfully utilised for studies of modifiers of cancer risk of rare genetic mutations (e.g., Andrieu *et al*, 2006; Antoniou *et al*, 2008; Win *et al*, 2011). Familial correlation in the risk of CRC was also accounted for using a robust variance estimation to derive appropriate measure of HR estimate imprecision (Rogers, 1993; Williams, 2000). Another strength was the standardised and uniformly high-quality testing for MMR gene mutations by the Colon CFR (Newcomb *et al*, 2007).

Potential limitations of this study include the use of self-reported weight and height and the often long period between age 20 years and the interview, meaning that participants often had to recall weight many years in the past. However, other studies have shown that measures of weight taken at age 20–30 years are well correlated with recalled values at age 50–70 years, with correlation coefficients ranging between 0.73 and 0.95 (Stevens *et al*, 1990; Perry *et al*, 1995; Troy *et al*, 1995; Norgan and Cameron, 2000; Tamakoshi *et al*, 2003). There is a possibility of response bias, where CRC-affected subjects recall their weight at age 20 years differently than unaffected subjects. It is also possible that the recall of weight at age 20 years may have depended on the methods of data collection, which varied by recruitment centre, and, therefore, may have resulted in decreased precision of reported weight data. Since cases with poor survival were less likely to be included in this analysis (as they were unable to provide a blood sample for genetic testing and complete a questionnaire), there is a possibility of survival and selection bias if age at onset and survival of cases were related to BMI and/or mutation status. We acknowledge that exposures such as physical activity and meat consumption that we have not adjusted for in this analysis may be confounders for the observed associations.

One previous study investigated the role of BMI in colorectal neoplasms for MMR gene mutation carriers, but the focus of their study was colorectal adenomas (Botma *et al*, 2010). They reported that MMR gene mutation carriers who are overweight or obese (current BMI ⩾25 kg m^–2^) had higher risk of adenomas compared with those with normal BMI (<25 kg m^–2^) for men (HR: 8.72; 95% CI: 2.06–36.96), but not for women (HR: 0.75; 95% CI: 0.19–3.07). A case–control study of CRC cases who met the revised Bethesda or Amsterdam criteria for Lynch syndrome and population controls observed both increased BMI at age 20 years and current BMI were associated with increased CRC risk for men, but not for women (Campbell *et al*, 2007). In our study, we did not observe statistical evidence for a difference between men and women for associations of BMI with CRC risk. Consistent with our results, Slattery *et al* (2003) found that obese people with a family history of CRC had approximately two-fold increased risk of CRC compared with those with normal BMI for both sexes combined (odds ratio: 1.65, 95% CI: 1.01–2.73).

There have been two population-based case–control studies examining the association between BMI and CRC with and without MSI, a phenotype that indicates loss of MMR function somatically, but not necessarily due to inherited mutations. Approximately 50% of CRCs with MSI diagnosed before age 50 years arise in MMR gene mutation carriers and 12% of cases diagnosed at age ⩾50 years are MMR gene mutation carriers (Hampel *et al*, 2008). These studies found positive associations between current BMI and microsatellite stable tumours, but no association with MSI-high tumours (Slattery *et al*, 2000; Campbell *et al*, 2010). It is difficult to compare these findings with our results because MSI tumours include CRCs caused not by an inherited MMR gene mutation but by methylation of *MLH1* (Herman *et al*, 1998; Poynter *et al*, 2008), and, therefore, these previous studies are probably not generalisable to MMR gene mutation carriers. Using the population-based cases from the Colon CFR (the same source of data for this analysis), Campbell *et al* (2010) found some suggestion that obese MMR gene mutation carriers, compared with those with normal BMI, had an increased risk of CRC (odds ratio: 3.96; 95% CI: 0.59–26.48) when MSI-high was further stratified by known MMR gene mutation status. As evidenced by the broad CIs, however, the study had insufficient numbers (only 85 carriers) to adequately investigate the association between BMI and CRC due to MMR gene mutations.

We observed a statistically significant association between BMI in early adulthood and CRC risk for *MLH1* and *PMS2* mutation carriers, but not for *MSH2* and *MSH6* mutation carriers. However, the associations were not statistically different from each other (*P*=0.51). The proteins produced by *MSH2* and *MSH6* from the MutS*α* heterodimer are responsible for detecting DNA mismatches occurring during replication, whereas the proteins produced by *MLH1* and *PMS2* from the MutL*α* heterodimer are responsible for cleavage (Kadyrov *et al*, 2006; Hewish *et al*, 2010). The association we observed with *MLH1* and *PMS2* mutations is consistent with obesity affecting CRC risk by inducing a second hit in the remaining wild-type allele of *MLH1* or *PMS2*. It is also consistent with obesity being a tumour promoter that is more relevant to tumours induced by MMR defects due to mutations in the MutL*α* heterodimer.

In this study, as all non-carriers are relatives of mutation carriers and, therefore, in general matched for familial factors that may be risk factors for CRC, the two groups are comparable. Given that we observed similar associations of BMI with CRC risk for carriers and non-carriers, we can surmise that having a mutation in an MMR gene does not alter the degree of increased risk imposed by BMI in early adulthood. In contrast, we have shown, using the same analytical design and data source, that non-carrier women were 74% more likely to develop endometrial cancer for every 5 kg m^–2^ increase in BMI at age 20 (HR: 1.74; 95% CI: 1.27–2.37; *P*<0.001), while carriers were not more likely to develop endometrial cancer with increased BMI (Win *et al*, 2011). Given MMR mutation carriers are rare in the general population (Dunlop *et al*, 2000), the vast majority of the general population are non-carriers and, therefore, our finding for non-carriers will be generalisable to the general population. Our findings for non-carriers are consistent, in terms of direction, with previously published studies that observed a positive association between BMI in early adulthood and subsequent CRC risk for the general population (Le Marchand *et al*, 1992; Lee and Paffenbarger, 1992; Must *et al*, 1992; Russo *et al*, 1998).

The biologic mechanisms underlying an association between body size in early adulthood and increased risk of CRC are not well understood. One theory suggests that obesity, particularly central obesity, increases insulin resistance and hyperinsulinaemia (Giovannucci, 1995; Kahn and Flier, 2000) via alterations in the signalling of endogenous hormones, particularly not only insulin and insulin-like growth factors, but also steroid hormones, and possibly, adipocyte-derived factors such as leptin and adiponectin (Gunter and Leitzmann, 2006; John *et al*, 2006). High circulating concentrations of insulin and C-peptide, as well as type 2 diabetes, are associated with increased risk of CRC (Kaaks *et al*, 2000; Larsson *et al*, 2005). It is of interest that MMR defects frequently result in mutations of the insulin-like growth factor receptor (IGFR), which involves this same insulin signalling system (Duval and Hamelin, 2002). It is not known if the effect of obesity in MMR gene mutation carriers is related to IGFR mutational events.

Our results suggest that BMI in early adulthood is a potential modifier of CRC risk for carriers of an MMR gene mutation. Currently, the only methods available for MMR gene mutation carriers to reduce their risk for CRC are screening by colonoscopy (de Jong *et al*, 2006), prophylactic subtotal colectomy (Smith and Rodriguez-Bigas, 2009) and aspirin chemoprevention (Burn *et al*, 2009). Avoiding obesity in early adulthood might also reduce the risk of CRC for MMR gene mutation carriers.

## Figures and Tables

**Table 1:** Baseline characteristics of study subjects (MMR gene mutation carriers and non-carriers)

	**Mutation carriers**		**Non-carriers**	
	**CRC-affected (*N*=659) number (%)**	**CRC-unaffected (*N*=665) number (%)**	**CRC-affected (*N*=36) number (%)**	**CRC-unaffected (*N*=1183) number (%)**
*Sex*				
Female	332 (50)	405 (61)	19 (53)	693 (59)
				
Age (year)^a^, mean (s.d.)	44.0 (11.1)	44.9 (13.7)	56.8 (14.8)	51.9 (15.6)
				
*MMR gene mutated*				
*MLH1*	273 (41)	227 (34)		
*MSH2*	308 (47)	340 (51)		
*MSH6*	40 (6)	77 (12)		
*PMS2*	38 (6)	21 (3)		
				
*Country of recruitment*				
Australia or New Zealand	355 (54)	484 (72)	16 (44)	967 (81)
USA	216 (33)	151 (23)	15 (42)	184 (16)
Canada	88 (13)	30 (5)	5 (14)	32 (3)
				
*Source of recruitment*				
Clinic based	473 (72)	599 (90)	23 (64)	1087 (92)
Population based	186 (28)	66 (10)	13 (36)	96 (8)
				
*Cigarette smoking* b				
Never	319 (49)	363 (55)	15 (44)	652 (55)
Ever	338 (51)	299 (45)	19 (56)	527 (45)
Unknown	2	3	2	4
				
*Alcohol consumption* c				
Never	233 (36)	193 (31)	16 (44)	417 (36)
Ever	408 (64)	439 (69)	20 (56)	732 (64)
Unknown	18	33	0	34
				
*Body size, mean (s.d.)*				
Height (cm)	170.1 (10.1)	169.8 (9.7)	169.7 (9.5)	169.4 (9.9)
Weight at age 20 (kg)	66.9 (15.5)	65.4 (14.6)	65.8 (13.0)	64.6 (13.8)
BMI at age 20 (kg m^–2^)^d^	23.0 (4.0)	22.5 (3.7)	22.7 (3.3)	22.4 (3.7)

Abbreviations: BMI=body mass index; CRC=colorectal cancer; MMR=mismatch repair.

a Age at first diagnosis of CRC for affected subjects; age at first polypectomy or diagnosis of another cancer or last contact for unaffected subjects (whichever came first).

b Cigarette smoking status at age 20 years; cigarette smoking was defined as ever smoking one cigarette per day for 3 months or longer.

c Alcohol consumption status at age 20 years; alcohol beverages include beer, wine, cider, spirits, mixed drinks or cocktails.

d Calculated from self-reported height and weight at age 20 years.

**Table 2:** Source of colorectal cancer diagnoses verifications and tumour characteristics

	**Carriers number (%)**	**Non-carriers number (%)**
*Source of verification*		
Pathology review/report	569 (86)	25 (69)
SEER/other cancer registry	17 (3)	0 (0)
Hospital or clinic record	9 (1)	1 (3)
Self/relative	64 (10)	10 (28)
		
*Tumour site*		
Right^a^	356 (61.1)	15 (58)
Left^b^	119 (20.4)	6 (23)
Rectosigmoid	30 (5.1)	2 (8)
Rectum	78 (13.4)	3 (11)
Unknown	76	10
		
*Histology grade*		
Well differentiated	46 (10)	1 (5)
Moderately differentiated	279 (62)	14 (74)
Poorly differentiated	124 (28)	4 (21)
Unknown	210	17
		
*AJCC stage* c		
I	122 (28)	4 (2.5)
II (A, B, C)	214 (50)	9 (53)
III (A, B, C)	94 (22)	4 (23.5)
Unknown	229	19
		
*Synchronous*		
Yes	44 (12)	2 (11)
No	313 (88)	17 (89)
Unknown	302	17

Abbreviation: AJCC=American Joint Committee on Cancer.

a Right colon included caecum, ascending colon, hepatic flexure and transverse colon.

b Left colon included splenic flexure, descending colon and sigmoid colon.

c Metastasis status was unavailable; and any of this stage could be stage IV.

**Table 3:** Hazard ratios for association between BMI at age 20 years and CRC risk for MMR gene mutation carriers and non-carriers

	**Total number**	**Total person-years**	**CRC number (%)**	**Univariable analysis**		**Multivariable analysis**	
				**HR (95% CI)^a^**	***P***	**HR (95% CI)^a^^,^^b^**	***P***
*Carriers*							
BMI (per 5 kg m^–2^)	1324	58 868	659 (50)	1.33 (1.11–1.58)	0.002	1.30 (1.08–1.58)	0.007
							
*WHO classification*							
Underweight	121	5353	54 (45)	0.91 (0.60–1.38)		1.04 (0.66–1.64)	
Normal	909	41 014	450 (50)	1.00 (referent)		1.00 (referent)	
Overweight	233	9971	118 (51)	1.17 (0.83–1.63)	0.01	1.12 (0.78–1.62)	0.06
Obese	61	2530	37 (61)	2.40 (1.35–4.27)		2.35 (1.30–4.23)	
							
*Non-carriers*							
BMI (per 5 kg m^–2^)	1219	63 436	36 (3)	1.86 (1.24–2.77)	0.002	1.64 (1.02–2.64)	0.04
							
*WHO classification*							
Underweight	113	6157	3 (3)	0.81 (0.23–2.88)		1.01 (0.28–3.73)	
Normal	904	47 641	25 (3)	1.00 (referent)		1.00 (referent)	
Overweight	158	7694	6 (4)	3.28 (1.09–9.84)	0.01	3.62 (1.05–12.55)	0.05
Obese	44	1944	2 (5)	4.98 (1.17–21.23)		3.00 (0.60–14.97)	

Abbreviations: BMI=body mass index; CI=confidence interval; CRC=colorectal cancer; HR=hazard ratio; MMR=mismatch repair.

a With robust variance estimation for familial correlation in risk.

b Adjusted for sex, country, cigarette smoking and alcohol drinking in both carriers and non-carriers; and further adjusted for specific MMR gene mutated in carriers.

**Table 4:** HR for association between BMI (per 5 kg m^–2^) at age 20 years and CRC risk separately for carriers of mutation in each MMR gene

**MMR gene mutated**	**Total number**	**Total person-years**	**CRC cases number (%)**	**Median age of diagnosis (range)**	**HR (95% CI)^a^**	***P***
*MLH1*	500	21 449	273 (55)	41 (17–73)	1.36 (1.04–1.77)	0.03
*MSH2*	648	28 560	308 (48)	44 (17–91)	1.28 (0.96–1.70)	0.09
*MSH6*	117	5870	40 (34)	50 (22–72)	0.84 (0.38–1.80)	0.62
*PMS2*	59	2989	38 (64)	49 (30–74)	1.52 (0.48–4.85)	0.48
*MLH1* and *PMS2* (MutL*α* heterodimer)	559	24 438	311 (56)	43 (17–74)	1.36 (1.05–1.76)	0.02
*MSH2* and *MSH6* (MutS*α* heterodimer)	765	34 430	348 (45)	45 (17–91)	1.26 (0.96–1.65)	0.09

Abbreviations: BMI=body mass index; CI=confidence interval; CRC=colorectal cancer; HR=hazard ratio; MMR=mismatch repair.

a adjusted for sex, country, cigarette smoking and alcohol drinking with robust variance estimation for familial correlation in risk.
