# Supplementary material for: Body mass index in early adulthood and colorectal cancer risk for carriers and non-carriers of germline mutations in DNA mismatch repair genes
Source: Br J Cancer. 2011 May 10;105(1):162–9. doi: 10.1038/bjc.2011.172 (PMC3137400; doi:10.1038/bjc.2011.172)
Supplement: Supplementary Information [file bjc2011172x1.doc]

Table S1 Number and weights applied to CRC-affected and -unaffected carriers of a germline mutation in a mismatch repair gene

| Age Group^*^ (year) | No of CRC-affected | No of CRC-unaffected | Person-year of CRC-affected | Person-year of CRC-unaffected | Population  incidence of CRC^†^ | age-specific incidence of CRC for carriers^‡^ | weight for CRC-affected | weight for CRC-unaffected |
| --- | --- | --- | --- | --- | --- | --- | --- | --- |
| 18-24 | 23 | 33 | 81 | 114 | 0.00003 | 0.00344 | 1.3568 | 0.7513 |
| 25-29 | 38 | 49 | 89 | 122 | 0.00005 | 0.00484 | 0.7791 | 1.1713 |
| 30-34 | 74 | 81 | 176 | 159 | 0.00008 | 0.00511 | 0.3761 | 1.5700 |
| 35-39 | 89 | 81 | 194 | 153 | 0.00013 | 0.00599 | 0.3102 | 1.7579 |
| 40-44 | 133 | 102 | 283 | 213 | 0.00018 | 0.00590 | 0.1595 | 2.0959 |
| 45-49 | 109 | 95 | 225 | 177 | 0.00030 | 0.00712 | 0.1613 | 1.9624 |
| 50-54 | 85 | 76 | 160 | 158 | 0.00050 | 0.00836 | 0.1586 | 1.9411 |
| 55-59 | 50 | 58 | 96 | 97 | 0.00088 | 0.01029 | 0.1899 | 1.6983 |
| 60-64 | 33 | 33 | 52 | 67 | 0.00135 | 0.01117 | 0.1832 | 1.8168 |
| 65-69 | 10 | 18 | 21 | 38 | 0.00215 | 0.01252 | 0.4120 | 1.3267 |
| 70-79 | 15 | 39 | 46 | 233 | 0.00300 | 0.01230 | 0.2554 | 1.2864 |

^*^ Age at first diagnosis of colorectal cancer (CRC) for CRC-affected subjects; age at first polypectomy or diagnosis of another cancer or last interview for unaffected subjects (whichever came first)

^†^ Population incidence rates of CRC averaged for males and females

^‡^ Age-specific incidences of CRC per year for MMR gene mutation carriers were obtained from (Pan*de et* al, 2010); Calculated as log-linear decay in relative risk (from 137.5 at age 18 to 4.1 at age 70) times Population incidence of CRC^†^

**References**

Pande M, Lynch PM, Hopper JL, Jenkins MA, Gallinger S, Haile RW, LeMarchand L, Lindor NM, Campbell PT, Newcomb PA, Potter JD, Baron JA, Frazier ML, Amos CI (2010) Smoking and Colorectal Cancer in Lynch Syndrome: Results from the Colon Cancer Family Registry and The University of Texas M.D. Anderson Cancer Center. *Clinical Cancer Research* **16**(4)**:** 1331-1339
